# Effects of RNAi-by-feeding on DNA methyltransferases in *Daphnia pulex*

**DOI:** 10.1093/eep/dvag022

**Published:** 2026-06-22

**Authors:** Trenton C Agrelius, Krista Harmon, Allison Reed, Samuel B Burnett, Rekha C Patel, Jeffry L Dudycha

**Affiliations:** Department of Biological Sciences, University of Notre Dame, Notre Dame, IN 46556, United States; Department of Biological Sciences, University of South Carolina, Columbia, SC 29208-0001, United States; Department of Biological Sciences, University of South Carolina, Columbia, SC 29208-0001, United States; Department of Biological Sciences, University of South Carolina, Columbia, SC 29208-0001, United States; Department of Biological Sciences, University of South Carolina, Columbia, SC 29208-0001, United States; Department of Biological Sciences, University of South Carolina, Columbia, SC 29208-0001, United States

**Keywords:** RNAi, *daphnia*, DNMT, immune response, epigenetics

## Abstract

DNA methyltransferases (DNMTs) are well-characterized epigenetic enzymes responsible for transferring methyl groups to and from DNA. Three main DNMT orthologues differ in function and methylation capability. They each are evolutionarily conserved across diverse taxa, but few studies investigate them jointly. We did so in *Daphnia*, freshwater microcrustaceans used extensively in research on genetic diversity, phenotypic plasticity, and maternal effects. *Daphnia* are capable of reproducing asexually through cyclic parthenogenesis, making the *Daphnia* system an ideal choice for studying epigenetic phenomena. Advances in gene expression control techniques, including RNA interference (RNAi), have increased the versatility and power of the *Daphnia* system. RNAi is a post-transcriptional gene silencing mechanism that operates through sequence-specific cleavage of endogenous messenger RNA transcripts. Here, we used an RNAi bacterial feeding regime to target the three DNMT genes in two clones of *Daphnia pulex*. We observed significant genotypic differences in response to the RNAi feeding regime, namely, the mortality of one clone. In the other, DNMT expression significantly increased in five of the six experimental treatments, with the highest level observed in animals treated with the GFP double-stranded RNA bacterial vector control. However, DNMT expression was reduced in all three DNMT RNAi treatments relative to the GFP control. Furthermore, we found strong cross-reactivity, where targeting one DNMT resulted in a reduction in expression of the other two. This response may be associated with known immune pathways involving signal transduction that can be stimulated by viral and bacterial signals, or it may result from previously unknown aspects of DNMT biology.

## Introduction

DNA methyltransferases (DNMTs) are epigenetic enzymes that add methyl groups (−CH3) to the 5′-carbon in cytosines (Goll and Bestor 2005). There are at least three well-characterized orthologues that differ in function and methylation capability. DNA methyltransferases 1 (DNMT1) is a highly conserved methyltransferase gene that maintains DNA methylation patterns across the genome by preserving DNA methylation after replication events [1]. DNMT3 methylates cytosines in a *de novo* fashion [1] and has been shown to respond to environmental stimuli [reviewed in 2]. Despite possessing all the conserved amino acid residues necessary for DNA methylation, DNMT2 proteins target and methylate tRNA molecules [reviewed in 3]. Intriguingly, DNMT2 is the only methyltransferase found in *Drosophila*, methylating DNA [3] at uncharacteristically low rates for animals [4]. DNMT2 is separated from the other DNMT clades phylogenetically [5], and it does not cluster with classic RNA-methyltransferases. DNMT2 is the most widely distributed methyltransferase, and it is generally thought that DNMTs evolved from a DNMT2-like ancestor [6]. Evidence suggests that DNMT3 has undergone multiple duplication events in several taxa [7], resulting in paralogs like DNMT3a, b, or L found in human, mouse, zebrafish [7], and more recently *Daphnia* [8, 9]. Despite the known relationships between the DNMTs and their functions, most studies do not study them jointly.

*Daphnia* (Class: Crustacea, suborder: Cladocera) are freshwater microcrustaceans that are used as a model organism in the fields of evolution, ecology, genomics, phenotypic plasticity, and, more recently, epigenetics [9–17]. *Daphnia* studies show high levels of phenotypic plasticity in response to a variety of biotic and abiotic environmental cues [18, 19] both within and across generations [17, 20–23]. Changes in gene expression, DNA methylation, and protein structure have been observed several generations removed from the original cue experienced by the mother [15, 24, 25].

*Daphnia* are cyclical parthenogens, usually reproducing asexually [26, 27], making *Daphnia* an ideal model system for studying transgenerational phenomena that reflect underlying epigenetic processes. Furthermore, the publication of high-quality genomes [13, 28] and advances in gene expression control techniques like viral transgenesis [29], CRISPR [30, 31], TALEN [32], and RNA interference (RNAi) [33–35] increase the versatility and power of the *Daphnia* system for understanding epigenetic processes.

The *Daphnia* genome contains all three DNMTs, with at least two DNMT3 paralogs [9], and *Daphnia* have been shown to alter DNA methylation within and across generations in response to heavy metal exposure [10, 11], salinity [19], radiation [36], and caloric or dietary restrictions [8, 37]. Furthermore, DNMT expression can be influenced by dietary restriction and is subject to maternal influence in clonal lineages [17]. Recent studies have shown that *Daphnia* respond to environmental stressors by altering the DNA methylation of their offspring in a stable, heritable manner relative to the current environment [38]. This transmission could alter the phenotypic trajectory of offspring as an anticipatory maternal effect, examples of which are common in *Daphnia* [18, 20, 39, 40].

RNAi is a post-transcriptional gene silencing mechanism that operates through sequence-specific cleavage of endogenous messenger RNA (mRNA) transcripts. The RNAi pathway is highly conserved across eukaryotes and has been used for loss-of-function studies in various model systems [41]. The RNAi pathway is activated by double-stranded RNA [42], complementary to the mRNA of a gene of interest. The dsRNA is targeted and cleaved into 21–24 nt, single-stranded, short-interfering RNAs that act as guides for the degradation of complementary mRNA transcripts but can also induce unintended changes in nontargeted proteins [43].

RNAi methods for gene knockdowns are well-established for several organisms [44–46], including *Daphnia* [33–35]. Schumpert et al. [35] developed a novel and efficient method to achieve RNAi in *Daphnia* through a daily bacterial feeding regime that potentially allows for the restoration of expression of the gene of interest by stopping the RNAi treatment. It eliminates the logistical challenges of microinjection-based approaches and allows for partial knockdown of genes for which complete loss of expression could be fatal.

The utility of a technique that allows for the return of function is in its ability to permit age-specific gene knockdown of most genes, including those that might prove lethal if knocked down during early developmental stages. DNA and histone methylation are essential elements for controlling gene expression, regulating developmental cycles, and determining cell fate [47–49]. Loss of a key epigenetic regulator like DNMT1 can lead to increased cell [50] and embryonic mortality [51] in vertebrate systems, making knockout studies increasingly difficult.

We previously found significant differences in DNMT expression between two clones of *D. pulex*, both within- and across-generations, and significant maternal effects in response to food limitation [17]. Data connecting differential DNA methylation to environmental stimuli are mounting [e.g. 19, 36, 52], but few studies have investigated the loss-of-function of the epigenetic regulators themselves [9]. We aim to use the RNAi feeding regime to individually knockdown the expression of three DNMTs in the *D. pulex* clones MORG 5 and TRO 3. By using the RNAi feeding regime, we hypothesize that DNMT expression can be reduced in the maternal generation (*G*_0_) to negligible levels without incurring high embryonic mortality rates (*G*_1_–*G*_2_). This would allow for mechanistic investigations into maternal (*G*_0_) and transgenerational (*G*_1_ and beyond) epigenetic changes.

## Results

### Construct sensitivity study

**Figure 1:**
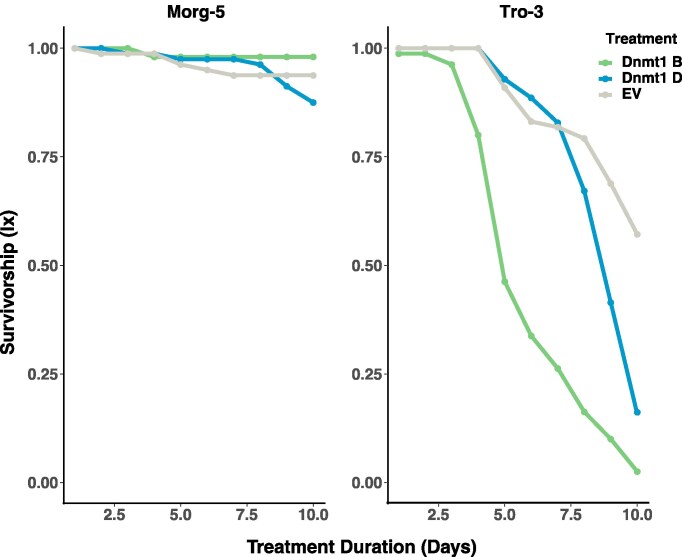
Plots showing the effects of each vector type on survivorship (lx) of the two clones, MORG5 and TRO3. Animals treated with the DNMT1 B vector are shown in green, DNMT1 D vector in blue, and EV in gray. No significant difference was observed between either DNMT vector when compared to EV in MORG5 (χ^2^= 0.142, *P* = .931, *df* = 2). 98% of MORG5 DNMT1 B, 76% of DNMT1 D, and 94% of EV survived the 10-day trial. DNMT1 treatment significantly reduced survivorship in TRO3 when compared to EV (χ^2^ = 60.974, *P* < .0001, *df* = 2). Death was 3 times as likely to occur when treated with DNMT1 B (*P* < .0001, *HR* = 3.765). There was no significant increase in mortality between DNMT1 D and EV (*P* < .0966, *HR* = 1.328). 2.5% of TRO3 DNMT1 B, 16% of DNMT1 D, and 60% of EV survived the 10-day trial.

MORG 5 survivorship was not significantly reduced by DNMT1B or DNMT1D RNAi vector treatments compared to animals fed bacteria with empty vectors (EVs), Fig. 1 (*χ*^2 ^= 0.142, *P* = .931, *df* = 2). TRO 3 survivorship was significantly reduced by RNAi vector treatments (*χ*^2 ^= 60.974, *P* < .0001, *df* = 2), with the DNMT1B RNAi vector increasing the probability of death by three times that of the EV (Fig. 1; *P <* .0001, *hazard ratio* = 3.765, *CI* = 2.694–5.260). The number of offspring produced per living female was reduced in all three treatments (DNMT1 B/D; EV) in both clones when compared to the maternal generation (MORG 5: *F*_3,23_ = 4.506, *P* < .012; TRO 3: *F*_3,13_ = 9.479, *P* < .001) but not between RNAi vector treatments (Supplemental Fig. 4). Fecundity did not significantly differ between MORG and TRO in the maternal generation, *G*_0_, used to produce experimental animals, *G*_1_.

Neonates, *G*_2_, produced during the *Construct Sensitivity Study* vector treatments from both clones were collected and treated with the same RNAi vector as their mother, *G*_1_. In both DNMT RNAi vector treatments, neonates experienced at least 50% mortality before reaching maturation. All TRO 3 offspring that survived developed a brood chamber (Supplemental Fig. 6) but did not begin the process of vitellogenesis during a 12-day bacterial feeding regime. Body pigmentation was within a normal range, and gut coloring remained a vibrant or pale yellow–green (Supplemental Fig. 6). MORG 5 neonates from the DNMT1D and EV treatments were observed to have completed the process of vitellogenesis on the 10th and 11th days of the 12-day trial. Embryo development appeared normal. Pigmentation and gut coloring resembled that of normal, well-fed *Daphnia*. Fat deposits were observed in neonates from both clones.

Photographs were taken after ten consecutive days of the RNAi vector treatment (Supplemental Fig. 5). Notable differences in animal body and gut coloring, presence or absence of vitellogenesis, offspring production, and carapace abnormalities were observed between the two genotypes. Note, we had not anticipated observing these kinds of characteristics, and thus our observational comparisons are informal because the photographs were not standardized. For all three RNAi vectors, gut coloring ranged from dark brown to clear in TRO 3 animals. Surviving animals from the DNMT1D RNAi vectors developed a “curly” phenotype in which deformities in the carapace impede or curl into the brood chamber. Vitellogenesis was not observed for the majority of the TRO 3 animal cohorts. In contrast, MORG 5 animals generally appeared less translucent, with gut coloring ranging from a vibrant green to a bright yellow. Vitellogenesis was commonly observed among all three vector treatments, and embryo development was generally unimpeded by the bacterial feeding regime.

Due to the adverse effects induced by the vectors on the TRO 3 clone in the *Construct Sensitivity Study*, we only used the MORG 5 clone to examine the potential effects of DNMT RNAi further. We did, however, test the effects of nonendogenous dsRNA on TRO 3 on survivorship in the *DNMT Comparison Study* (Supplemental Fig. 7). GFP-dsRNA (GFP) vectors significantly reduced TRO 3 survivorship (χ^2 ^= 14.046, *P* < .029, *df* = 6), with only ten of thirty TRO 3 animals surviving a 10-day trial. Remaining TRO 3 animals appeared translucent, with no pigmentation in the carapace outside the compound eye and little to no coloring in the gut (Supplemental Fig. 8B). Despite reaching maturation before the experiment’s start, no offspring were released from the brood chamber. Additional vitellogenesis events were not observed during the GFP bacterial treatment.

### DNMT comparison study: RNAi vector effects on phenotypic traits

**Figure 2:**
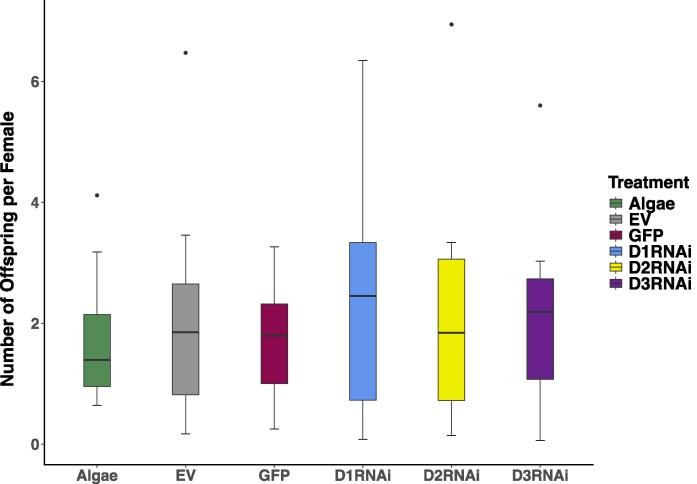
Boxplots of the number of offspring produced by the MORG5 clone. Values were standardized by dividing the total number of neonates produced each day by the total number of surviving females. Treatment identification is as follows: algae (green), EV (gray), L4440 with GFP (turquoise), and each DNA methyltransferase RNAi treatment 1 (blue), 2 (yellow), and 3 (purple). No significant difference was detected between treatments (*F*_5,42_ = 0.024, *P* = .997, *df* = 5). TRO3 animals in the GFP plasmid vector did not produce offspring during the 10-day bacterial feeding regime and were not included in the figure.

MORG 5 survivorship was not significantly reduced by RNAi vector treatments (*χ*^2 ^= 0.366, *P* < .996, *df* = 5; Supplemental Fig. 9). No significant difference in the number of offspring per female was detected between the MORG 5 RNAi vector treatments (*F*_5,42 _= 0.024, *P* = .997, *df* = 5; Fig. 2).

Within 18 h of receiving the first vector dose, MORG 5 animals in the D1RNAi, D2RNAi, and D3RNAi treatments prematurely shed their carapace. After the second vector dose, animals from the same treatments molted again. We did not observe similar events in the Algae, EV, or GFP controls. Photographs from each treatment on day 10 showed distinct differences in animal pigmentation, gut coloring, and reproduction (Supplemental Fig. 8). MORG 5 animals from the Algae, EV, and GFP treatments showed normal pigmentation and gut coloring ranging from pale to bright green, indicating continuous feeding on *Ankistrosdesmus falcatus* (green algae) and presumably bacteria. Animals from DNMT RNAi treatments showed abnormal pink pigmentation, suggesting elevated levels of hemoglobin. Gut coloring appeared a bright green. Continued vitellogenesis was observed for all MORG 5 animals, and embryo development appeared normal.

### DNMT comparison study: RNAi vector effects on gene expression

**Figure 3:**
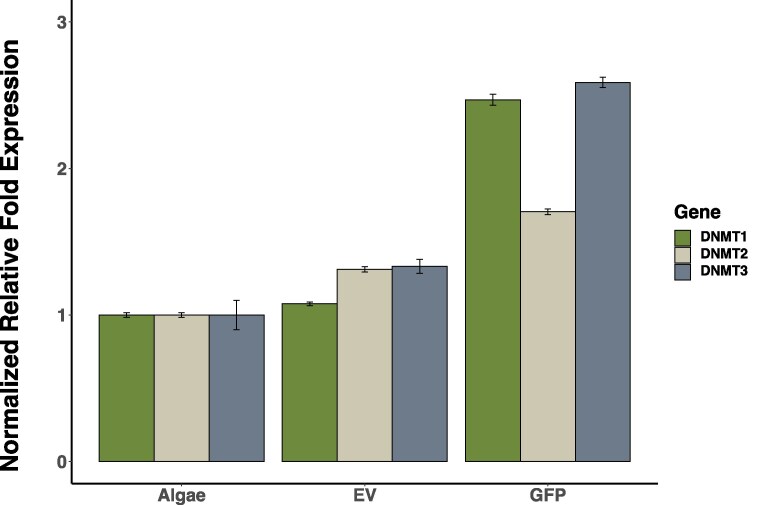
DNMT expression relative to XBP1 and normalized to the Algae control. EV treatments significantly increased DNMT2 and 3 expression. dsRNA (GFP) significantly increased mRNA transcripts for all three methyltransferase genes.

**Figure 4:**
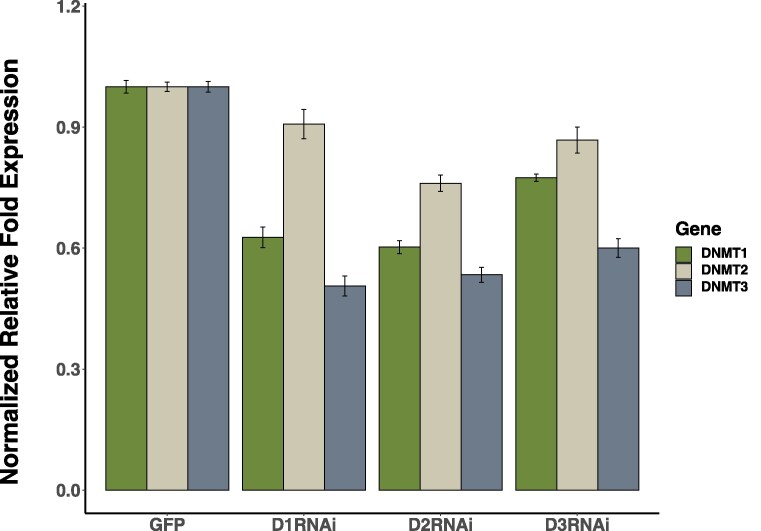
DNMT expression relative to XBP1 and normalized to the GFP control. Note that names for each DNMT RNAi vector treatment were abbreviated to conserve space. Nomenclature is as follows: D1 refers to the DNMT1 RNAi vector treatment. Expression of nontarget methyltransferase genes also reduced in each RNAi treatment.

DNMT expression in the three controls used for the *DNMT Comparison Study* (Table 1) was scaled to the Algae control cohort (Fig. 3). Significant increases in DNMT expression were observed in both EV (DNMT2 and 3) and GFP (DNMT1, 2, and 3) treatments (Supplemental Tables 1, 2, 3). EV treatments increased DNMT2 and 3 expression by ∼0.3 fold; GFP treatments increased DNMT1, 2, and 3 expression by 1.47, 0.7, and 1.59 fold, respectively. DNMT expression was significantly reduced by DNMT RNAi vector treatments when scaled to the GFP control (Fig. 4; Supplemental Tables 1, 2, and 3). However, DNMT RNAi vector treatments did not reduce DNMT expression levels below the Algae control. We observed off-target DNMT expression reductions in each DNMT RNAi vector treatment (Fig. 4). The magnitude of the off-target reduction varied between RNAi treatments (Supplemental Fig. 10).

**Table 1:** List of the DNMT comparison study treatments.

Treatment	Information on and purpose of the treatment in the experiment
Algae control	Negative control, *ad libitium*, or high-food diet consisting of 20 000 cells per ml of *Ankistrosdesmus falcatus* algae
Empty vector control	Isolate the effects generated by increased bacterial concentrations on *Daphnia* life history and DNMT expression. Empty L4440 vector used to transform Ht115 *E. coli* cells
GFP-dsRNA (GFP) control	L4447 plasmid with 5′ half of the GFP ORF used to isolate the effects generated by the presence of double-stranded RNA species on life history and DNMT expression. GFP is a nonendogenous target, any effect observed would be attributed to the RNA species and/or increased bacterial concentrations
D1RNAi (D1)	L4440 plasmid with the PCR product from DNMT1; knockdown the expression of DNMT1
D2RNAi(D2)	L4440 plasmid with the PCR product from DNMT 2; knockdown the expression of DNMT2
D3RNAi(D3)	L4440 plasmid with the PCR product from DNMT 3; knockdown the expression of DNMT3

## Discussion

We found significant impacts of the L4440/HT115 RNAi bacterial feeding regime on DNMT gene expression and *Daphnia* biology. Although we were unable to measure gene expression in our first experiment, it helped us focus our second experiment on a single *D. pulex* clone that tolerated the RNAi procedure well. That experiment produced three intriguing findings regarding RNAi treatments and DNMT gene expression. First, DNMT expression in *D. pulex* clone MORG 5 significantly increased in response to being fed either the EV or the vector with the GFP RNAi construct compared to being fed only the Algae control. Second, all three DNMT RNAi treatments significantly reduced DNMT expression compared to GFP RNAi. Third, despite our efforts to ensure specific binding of our RNAi constructs to their respective DNMTs, targeting any one DNMT reduced mRNA levels of the other two DNMTs, although the off-target effects differed in magnitude.

Observing expression changes in DNMT3 and DNMT1 following exposure to the EV and GFP RNAi treatments was not unexpected. The greater than two-fold expression increase in the GFP RNAi treatment suggests these genes are substantial parts of the *Daphnia* response to the presence of double-stranded RNA. DNMT3 is a *de novo* methyltransferase capable of responding to environmental cues [1], while DNMT1 functions as a maintenance methyltransferase, preserving DNA methylation during replication events [1]. Pathogen exposure and infection, including *Escherichia coli*, have been shown to induce and repress DNMT activity [53] and induce differential methylation patterns across thousands of loci, resulting in extensive epigenetic remodeling and changes in gene expression [54, 55] capable of influencing immune responses [56].

Double-stranded RNA immune responses are very well characterized in vertebrates to induce gene silencing using an antiviral program mediated by Type 1 interferons [57] that have pleiotropic functions in both innate and adaptive immunity. Invertebrate genomes lack genes associated with the interferons; however, Robalino et al. [58] observed a robust antiviral immunity induced by dsRNA in marine shrimp (*Litopenaeus vannamei*, clade Eumalacostraca) and sequence-specific targeting and destruction of mRNA transcripts. Unintended consequences like stimulation of immune system responses [58] and nonspecific protein targeting via the siRNA produced in the RNAi pathway are well documented in other systems [59, 60, reviewed in 61, 62], and thus an siRNA-triggered immune response may have occurred here.

RNAi relies on triggering an innate immune response that destroys complementary nucleic acid targets. Our targets were mRNA transcripts from genes responsible for maintaining the stability of the genome through DNA methylation [63]. However, the elevated concentration of transformed bacteria compared to the Algae-only control may have initiated a separate immune response, resulting in the increased DNMT expression observed in the EV treatment. Bacterial-mediated immune responses in *Daphnia* rely on cell surface receptors from the TOLL gene family that function as signal transducers [64, 65]. TOLL proteins show specificity with interactions between bacterial strains and rely on several well-conserved genes for signal transduction [64, 66]. Orthologues of at least seven of those genes have been identified in *D. pulex* (TOLL, Myd88, Relish, Pelle, Cactus, Imd, STAT) and other immune-related genes that respond directly to bacteria [67]. The TOLL pathway has been identified as a vital component in the *Drosophila* antiviral response [68–70] and can be stimulated by *E. coli* as well as dsRNA in crustaceans [57, references within]. Immune responses to pathogens in *Daphnia* have been detailed as early as 1884 [71], making it plausible that the responses observed in our studies are due to an immune response outside the traditional RNAi-directed pathway. Future *Daphnia* epigenomic studies can test our hypothesis by tracking the expression of TOLL pathway genes when animals are challenged by transformed bacteria.

### Off-target effects of RNAi knockdown of DNMTs: nonspecific DNMT knockdown

Though extensive measures were taken to ensure that our RNAi constructs would target transcripts from only the gene of interest, we observed a reduction of non-targeted DNMT expression in all three of our DNMT RNAi treatments. The magnitude of the off-target expression reduction varied based on which DNMT was targeted (Fig. 4) and which off-target DNMT was observed. Crosstalk between epigenetic machinery is heavily documented [72, 73], resulting in changes in the expression of multiple epigenetic genes. The targeted reduction of one DNMT could impact the expression of another as part of DNMT biology, or an alternative mechanism could be facilitating the off-target reduction.

Within the RNAi machinery, the argonaute protein is guided by 20–24 nt siRNAs to cleave mRNA targets [reviewed by 74]. We ensured that each 300 nt methyltransferase amplicon cloned into L4440 vectors had no more than a 12 nt continuous alignment with mRNA from the other methyltransferase genes. This should eliminate the possibility of cross-reactivity mediated by the argonaute protein in the RNAi complex due to potential siRNAs lacking specificity. However, it is possible that the imperfect matches of the siRNA resulted in off-target translational repression and subsequent mRNA decay that is argonaute-independent. Endogenous microRNAs (miRNAs) can lead to translational repression by binding to mRNA, thereby preventing translation and facilitating exonucleolytic decay [reviewed by 75]. Only partial complementarity is needed between the miRNA and mRNA for the translational repression to occur [76]. This mechanism could explain the nonspecific reduction of methyltransferase gene expression.

### Insight into *Daphnia* biology: RNAi effects on fecundity, phenotype, and genotype

We expected to observe some genotypic differences between the two clones during the *Construct Sensitivity Study*. Previous work with the two clones had shown TRO3 to delay maturation, switch to resting egg production, and increase DNMT expression in response to reductions in resource quantity [17]. Still, the significant contrast in responses between the two clones was unexpected, especially in the EV treatment. Schumpert et al. [35] did not observe changes in reproduction for either *D. melanica* or the *D. pulex* genotype used, nor were there mortality rates exceeding 20%. Subsequent *Daphnia* RNAi studies report effective gene knockdowns without unexplained mortality in *D. pulicaria* [77], *D. magna* [78], *D. pulex* [79, 80], and *D. galeata* [81]. In fact, Lin et al. [79] reported offspring production was increased in RNAi-treated animals compared to controls. Thus, it seems clear that the extreme susceptibility of the TRO3 clone to RNAi-by-feeding is not the norm in *Daphnia*. Despite this, future RNAi-by-feeding studies should consider clonal variability and the potential for genetic background to alter results, along with the possibility that some clones may simply be intolerant of the procedure.

Visible signs of stress were observed during the *Construct Sensitivity Study* in the TRO3 animals. Signs included higher mortality rates, ranging from 40% to 97.5%, including the EV, where nearly half of the cohort died. We informally observed that all TRO3 animals reduced their filtering rates or stopped them completely, and their gut coloring shifted from green to brown. Filter-feeding in *Daphnia* is physically linked to respiration, with most of their oxygen supply coming from the internal current generated by the feeding combs’ movement [82]. By slowing or halting the filtering process, algal cells remain in the gut and decompose, resulting in a yellow or brown color; effectively, the animal loses the nutrient content of the food and starves while also experiencing hypoxia. This was observed in both DNMT1 and EV treatments of the TRO3 clone but not in MORG5. Rather, MORG5 seemed relatively unaffected by the vector treatments and continued producing offspring throughout the 10-day trial.

MORG5 animals in the *DNMT Comparison Study* experienced low mortality and showed no significant differences in offspring production among RNAi or control treatments ( Fig. 2), unlike the TRO3 animals, which produced no offspring when treated with GFP RNAi vectors. However, MORG 5 animals showed clear stress and potentially immune-related responses in all of the RNAi treatments (GFP and DNMT) that were not observed in the TRO3-GFP treatment, namely a startling double ecdysis event within 36 h of the bacterial treatment and high levels of apparent hemoglobin production. The different responses of TRO3 and MORG5 show that *Daphnia pulex* harbors major genetic differences in how animals respond to double-stranded RNA, despite otherwise normal life histories.

Targeting methyltransferase transcripts could be a primary cause for the observed premature molting in MORG5. Molting in juvenile *Daphnia* is controlled primarily through the hormone 20-hydroxyecdysone and its interaction with juvenile hormones [83, 84]. Molting results in a major loss of calcium: ∼90% of total body calcium is lost each molt [85]. This loss is not replaced through diet, but rather, calcium uptake is an active process from the environment [86]. Juvenile hormones respond to environmental cues, resulting in increased expression of hemoglobin [87], changes in morphology [88], and oogenesis [89]. We began feeding bacteria 6 days after birth, which is the average date of maturation for MORG5 [17]. At this early stage, developmental processes are still mediated through juvenile hormones and presumably susceptible to environmental cues. DNMT3 responds to environmental cues and establishes new methylation patterns across the genome, while DNMT1 maintains the existing methylation profile, especially during development [1]. Both genes are highly expressed during developmental processes, and methylation profiles across the genome show differential patterns [1]. Changes in expression for either methyltransferase during the first reproductive event could impact the ecdysis process, resulting in both the abortive event and molting being observed.

Our work provides an interesting and novel insight into DNMT and *Daphnia* biology. DNMT 1, 2, and 3 expression is rarely studied together, and our results suggest a potent crosstalk mechanism between the methyltransferases that warrants further investigation in joint studies. Future research can investigate several questions, including whether the RNAi effect on DNMTs can be fine-tuned to limit off-target effects. What is the relationship between RNAi, genotype, and mortality? Do the DNMT RNAi treatments alter DNA methylation or other epigenetic markers? Does RNAi influence maternal effects? Could the loss of methyltransferase activity limit plasticity, thereby reducing the variance observed in trait means responding to environmental stressors?

## Methods

### Design and construction of RNAi vectors

Primers were designed to amplify 300 bp products from each DNMT gene to be used in RNAi vectors. In an attempt to minimize off-target effects, potential amplicons were aligned to mRNA sequences from the other DNMT genes. Primer sets were then selected only if alignments were <12 consecutive nucleotides between the amplicon and non-target DNMT mRNAs (Supplemental Figs. 1, 2, and 3), and amplicons did not overlap with regions used for qPCR. Amplicon size was chosen to maximize the number of siRNAs produced from a single dsRNA product (300 bp double-stranded RNA cut into ∼25 nt single-stranded siRNAs totaling 24 potential guides for the RNAi machinery), generating a more robust RNAi response. Restriction enzyme sites for XbaI and NheI were engineered into the 5′ ends of selected primers to allow for subcloning between pGEM-T Easy and L4440 plasmids [90].

Experiment 1, referred to hereafter as the *Construct Sensitivity Study*, was initially designed to evaluate whether targeting different regions of a DNMT mRNA transcript would yield a more robust response. Thus, the *Construct Sensitivity Study* targeted two locations on DNMT1 (Fig. 7). We opted to include all three DNMTs in Experiment 2, referred to hereafter as the *DNMT Comparison Study*, and limited the target location to one unique position on each DNMT transcript (Fig. 7). Selected amplicons were cloned into pGEM-T Easy plasmids (Promega) and verified by sequencing. pGEM-T Easy plasmids are designed with 3′-T overhangs that allow unmodified PCR products with poly-A tails to be cloned directly into the plasmid. Transformations were carried out using competent DH5-α *E. coli* cells (New England Biolabs), and plasmid purifications were performed using a Qiagen Plasmid Miniprep kit. DNMT amplicons were cut out of pGEM-T Easy constructs using appropriate restriction enzymes and gel-purified for subcloning into L4440. Finally, glycerol stocks were made using 500 μl of transformed DH5-alpha bacteria with positive recombinant pGEMT Easy constructs and 500 μl of glycerol. Stocks were stored at −80°C.

**Figure 5:**
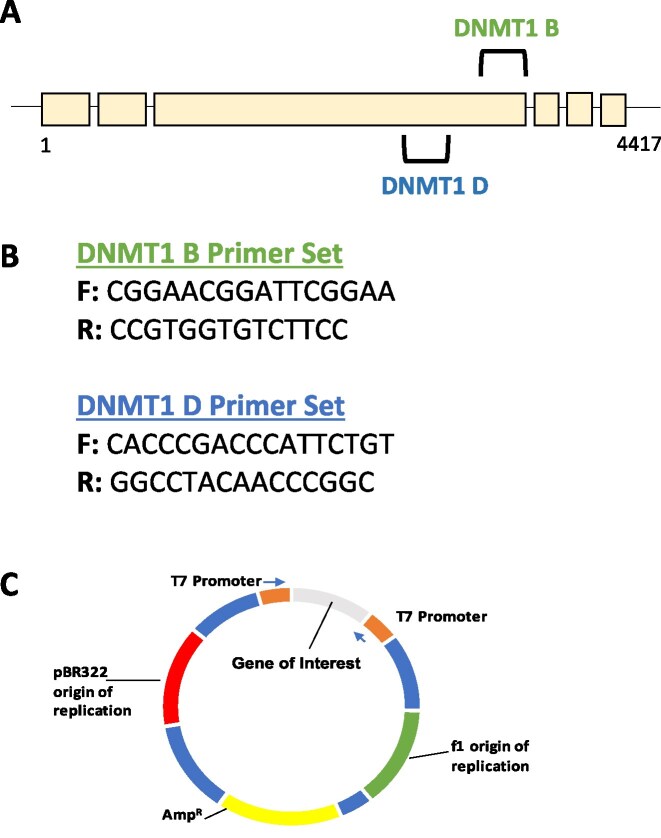
(A) *Daphnia pulex* DNMT1 gene schematic. Marked on the schematic are the two regions amplified by PCR and cloned into pGEMT-Easy and L4440. (B) Primer sets used to amplify 300 nucleotide regions of the DNMT1 gene. There is no overlap between the two amplicons. (C) Schematic diagram of the L4440 plasmid vector designed by Fire et al. [90]. The flanking T7 promoters (orange) can be induced to produce double-stranded RNA of the cloned insert from the gene of interest (gray).

Purified DNMT PCR products were subcloned into L4440 plasmid vectors (L4440 was a gift from Andrew Fire, Addgene plasmid #1654; http://n2t.net/addgene:1654; RRID:Addgene_1654). The L4440 vector allows for cloning PCR products between two T7 promoters oriented in opposing positions (Fig. 5). All L4440 plasmids were treated with calf intestinal phosphatase (CIP, New England Biolabs) prior to ligation reactions to increase ligation efficiency. Cell transformations were performed using HT115 (DE3) (W3110, rnc14:: DTn10 (Addgene), [91] *E. coli*. HT115 bacterial strains contain a source for T7 RNA polymerase (DE3 lysogen), which can be induced through the addition of 2 mM IPTG and are deficient in RNase III, leading to efficient production of dsRNA of the amplicon cloned between the two T7 promoters of L4440.

We set up a bacterial feeding regime [35] that exploits the RNAi pathway to induce endogenous gene knockdown in the *Daphnia* system. Transformed bacteria (bacteria with the plasmid L4440 containing an amplicon from a gene of interest) were grown overnight (∼14–16 h) in Luria Broth (LB) with 2 mM of IPTG to induce the production of dsRNA. The optical density (OD_600_) of bacterial cultures was measured using an Eppendorf BioPhotometer. Bacterial cultures from an OD_600_ of 2.8 units were pelleted and resuspended in 200 ml of filtered lake water to feed the bacterial culture to *Daphnia*, resulting in a final concentration of 0.014 OD_600_ or ∼1.2 × 10^7^  *E. coli* cells. New bacterial cultures were prepared daily using fresh LB and IPTG. In addition to the bacterial cells, *Daphnia* were fed quantitatively 20 000 cells/ml of *A. falcatus* daily, resulting in a mixture of bacteria and algae. New bacterial cultures were prepared daily throughout their administration to *Daphnia*

### Testing effects of DNMT RNAi vectors

Two experiments were conducted using the RNAi vectors and bacterial feeding regime. The *Construct Sensitivity Study* used two *D. pulex* clones, MORG 5 and TRO 3, and two RNAi vectors that targeted DNMT1. The *DNMT Comparison Study* targeted all three DNMT genes with unique RNAi vectors using the MORG 5 clone. The *D. pulex* clones originated from temporary ponds in Maine and Michigan, USA, respectively, and have been cultured under constant laboratory conditions to maintain continuous asexual reproduction. The clones have been previously characterized and observed to exhibit significant maternal effects, corresponding to changes in DNMT expression in response to diet [17]. All animals were propagated using filtered lake water (to 1 µm) collected locally from an outflow of Lake Murray in Lexington County, SC, USA, and maintained at 20°C under a 12:12 light: dark photoperiod. Laboratory-reared animals were fed a daily vitamin-fortified diet of the green alga *Ankistrodesmus falcatus* [92]. To minimize potential maternal effects-related variation in gene expression and epigenetic motifs [40], three acclimation generations were reared under standardized, high-food conditions. Animals in each generation were taken from the third clutch and fed a daily diet of 20 000 cells/ml of *A. falcatus*. Each animal was housed separately in a 150 ml beaker with 100 ml of filtered lake water and transferred every other day into a new beaker with fresh lake water. Each beaker was lightly dusted with cetyl alcohol to prevent entrapment on the water surface [93].

**Figure 6:**
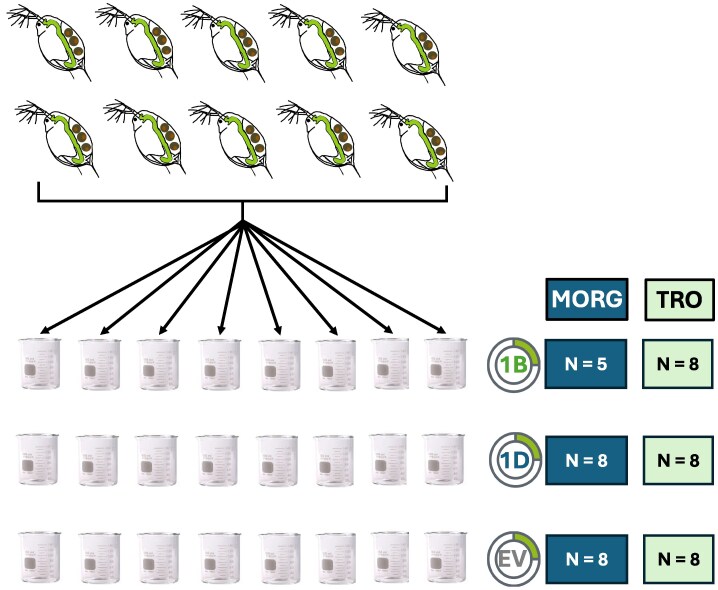
Construct Sensitivity Study schematic. Cohorts of ten adult female *Daphnia* were pooled into 250 ml beakers with eight replicate cohorts per treatment (1B, 1D, EV) per clone, except that MORG 1B had only five replicates.

**Figure 7:**
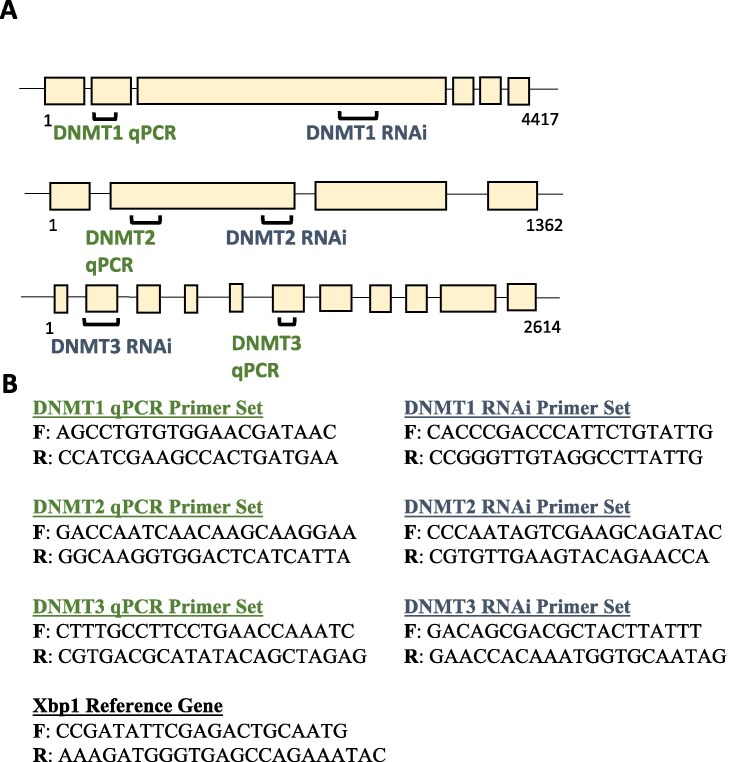
(A)  *Daphnia pulex* DNMT1, 2, and 3 gene schematics. Marked on the schematic are the two regions amplified by PCR for cloning into L4440 (RNAi) or qPCR. (B) Sequence information of the primer sets used to amplify 300 nucleotide regions of the DNMT genes (RNAi) and 100 nucleotide regions (qPCR). There is no overlap between the two amplicons. Xbp1 was used as a reference for gene expression analyses.

The *Construct Sensitivity Study* consisted of replicate cohorts of 10 female *Daphnia* per clone per 250 ml beaker with 200 ml of filtered lake water (Fig. 6). Eight replicate cohorts were randomly assigned to one of three treatments: EV, DNMT1B, or DNMT1D. DNMT1B and DNMT1D are RNAi vectors targeting different regions of DNMT1 mRNA transcripts (Fig. 7). For the MORG 5 DNMT1B treatment, we could only set up 5 replicate cohorts due to the lack of female neonates at the time of setup (total *n* = 450). Offspring born to females in each vector treatment during the experiment were collected and set up in cohorts of 10 under the same feeding regime as their mothers.

**Figure 8:**
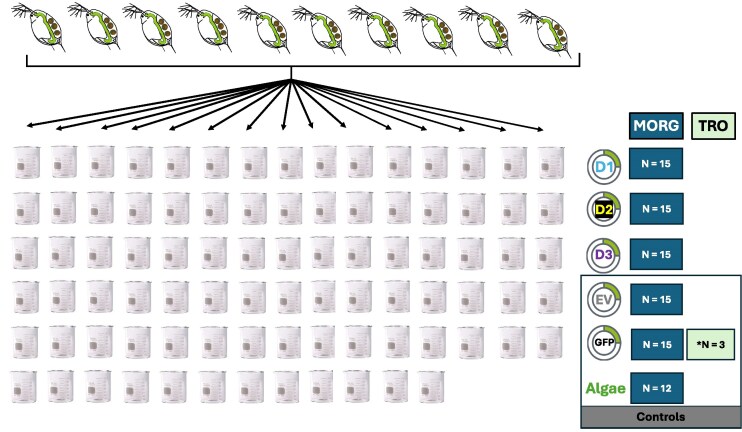
DNMT Comparison Study schematic. Cohorts of ten adult female *Daphnia* were pooled into 250 ml beakers with fifteen replicates of most treatments (D1RNAi, D2RNAi, D3RNAi EV, and GFP) in clone MORG. Only twelve replicated cohorts could be made for the Algae-only control. Three replicate cohorts of TRO were also included for the GFP control. The box indicates the three types of experimental controls.

The *DNMT Comparison Study* consisted of six treatments: three controls, and three RNAi DNMT treatments, which can be seen in Table 1. Three controls were necessary to (1) serve as a negative control and (2) partition out the effects of bacterial and dsRNA presence on DNMT gene expression. Replicate cohorts of 10 *Daphnia* per treatment in 250 ml beakers with 200 ml of filtered lake water were randomly assigned to one of the six feeding treatments (total of *n* = 870 experimental animals, Fig. 8). Most treatments consisted of 15 replicates, but we could only establish 12 replicate cohorts for the Algae-only control due to the lack of births during the experimental setup.

Bacterial feeding treatments for both experiments were initiated within 5–8 h of animals’ maturation (defined as eggs being deposited into the brood chamber) and continued for 10 days. During the 10-day bacterial feeding regime, we recorded mortality, the number of offspring produced per day (or the time to maturation for the offspring in the *Construct Sensitivity Study*), and several qualitative characteristics, such as gut color and vitellogenesis. Due to complications incurred from COVID-related shutdowns, animal tissue for qPCR gene expression work could not be collected for the *Construct Sensitivity Study*. Animal tissue was collected at the end of the 10-day trial in the *DNMT Comparison Study* and stored in RNAlater (Invitrogen) at 4°C for 4 days before being stored at −80°C until RNA extractions.

### Gene expression

For total RNA extractions, animals were pooled into cohorts (10 animals per treatment) for a single biological replicate (5 biological replicates per treatment per gene per clone). Prior to extraction, eggs were carefully dissected out of the brood chamber to exclude embryonic gene expression. Total RNA was then extracted from homogenized tissue using a Trizol/Purezol-Chloroform method (Purezol, Bio-Rad). Sample purity, concentration, and integrity were assessed using Nanodrop 2000, Quibit, and gel electrophoresis. gDNAase Iscript (Bio-Rad) was used to remove any genomic contamination and convert into first-strand cDNA.

Gene-specific qPCR primers were previously designed and validated for all three DNMTs [17]. Primer sequences and relative positioning compared to amplicons used for RNAi can be seen in Fig. 7. qPCR primers amplified nonoverlapping regions of each gene to ensure dsRNA produced by the plasmid vector treatment would not be detected. qPCRs were run with three technical replicates per biological sample using PowerUp SYBR Green Master Mix (Applied Biosystems) in a Bio-Rad CFX96 Real-Time PCR System (Bio-Rad). Thermal cycling conditions were: 2 min at 50°C and 2 min at 95°C, followed by 40 cycles of 15 s at 95°C and 30 s at 60°C. Dissociation curve analysis and gel electrophoresis were performed to confirm correct amplicon size and primer specificity. No-template controls and no-reverse-transcriptase controls were used to confirm the absence of contamination. Relative DNMT mRNA levels were normalized to XBP1 transcript level using the Pfaffl method, with an efficiency correction calculated from Real-time PCR Miner [94].

## Statistical analyses

Statistical analyses were conducted in R, version 3.6.2 [95], and plots were generated using ggplot2 [96]. Effects of the RNAi treatments on survivorship (*l_x_*), a type of ‘time to event’ data, were analyzed using a Cox proportional hazards regression with an Efron method to handle tied events. To test for the effects of each RNAi treatment on offspring production, the total number of offspring produced (per treatment) was divided by the total number of surviving mothers (per treatment) for each day. RNAi treatment effects on offspring data were analyzed using a type-III ANOVA. Effects of RNAi bacterial treatments on gene expression data were analyzed using a Kruskal–Wallis rank-sum test and a Dunn’s test for post hoc multi-comparisons. Data normality was tested using Shapiro–Wilks tests and visual comparisons; the number of offspring was log-transformed to meet the assumptions of normality. Data variance heterogeneity was assessed using Levene’s test.

## Supplementary Material

dvag022_Supplemental_File

## Data Availability

Data are available in Dryad: https://doi.org/10.5061/dryad.r2280gbrq.
