# Supplementary material for: Effects of RNAi-by-feeding on DNA methyltransferases in Daphnia pulex
Source: Environ Epigenet. 2026 Jun 22;12(1):dvag022. doi: 10.1093/eep/dvag022 (PMC13382199; doi:10.1093/eep/dvag022)
Supplement: dvag022_Supplemental_File [file dvag022_supplemental_file.docx]

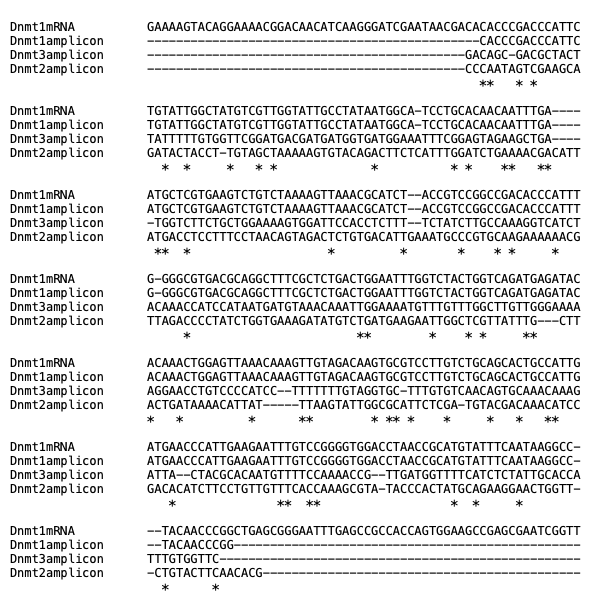


**Supplemental Figure 1.** Alignment between the mRNA transcript sequence of DNMT 1 and the amplicon sequences used for cloning into the L4440 plasmid.


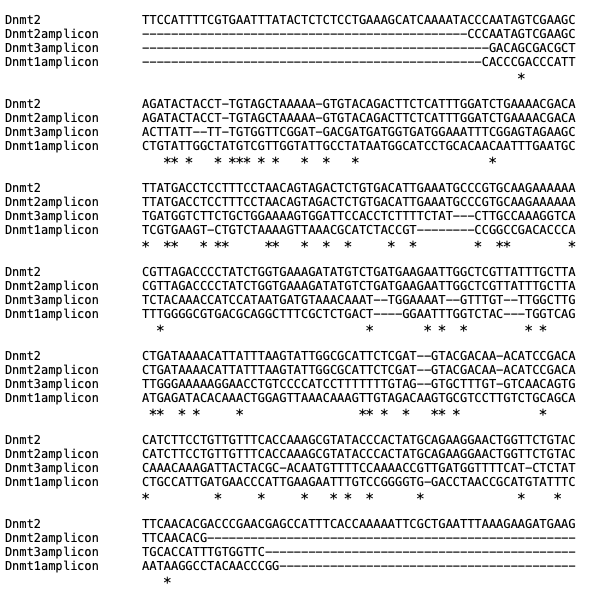


**Supplemental Figure 2.** Alignment between the mRNA transcript sequence of DNMT 2 and the amplicon sequences used for cloning into the L4440 plasmid.


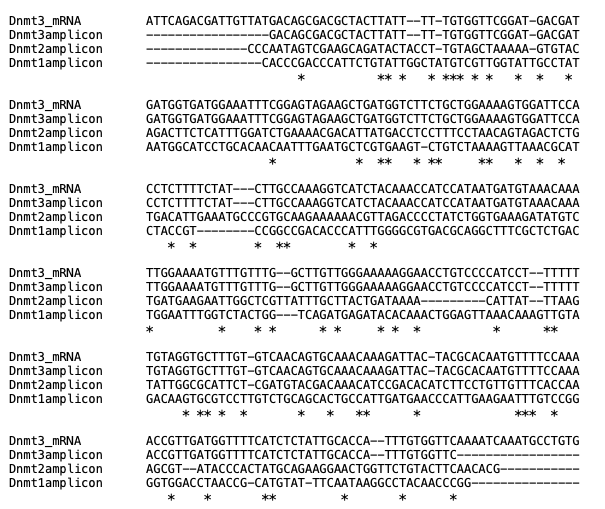


**Supplemental Figure 3.** Alignment between the mRNA transcript sequence of DNMT 3 and the amplicon sequences used for cloning into the L4440 plasmid.


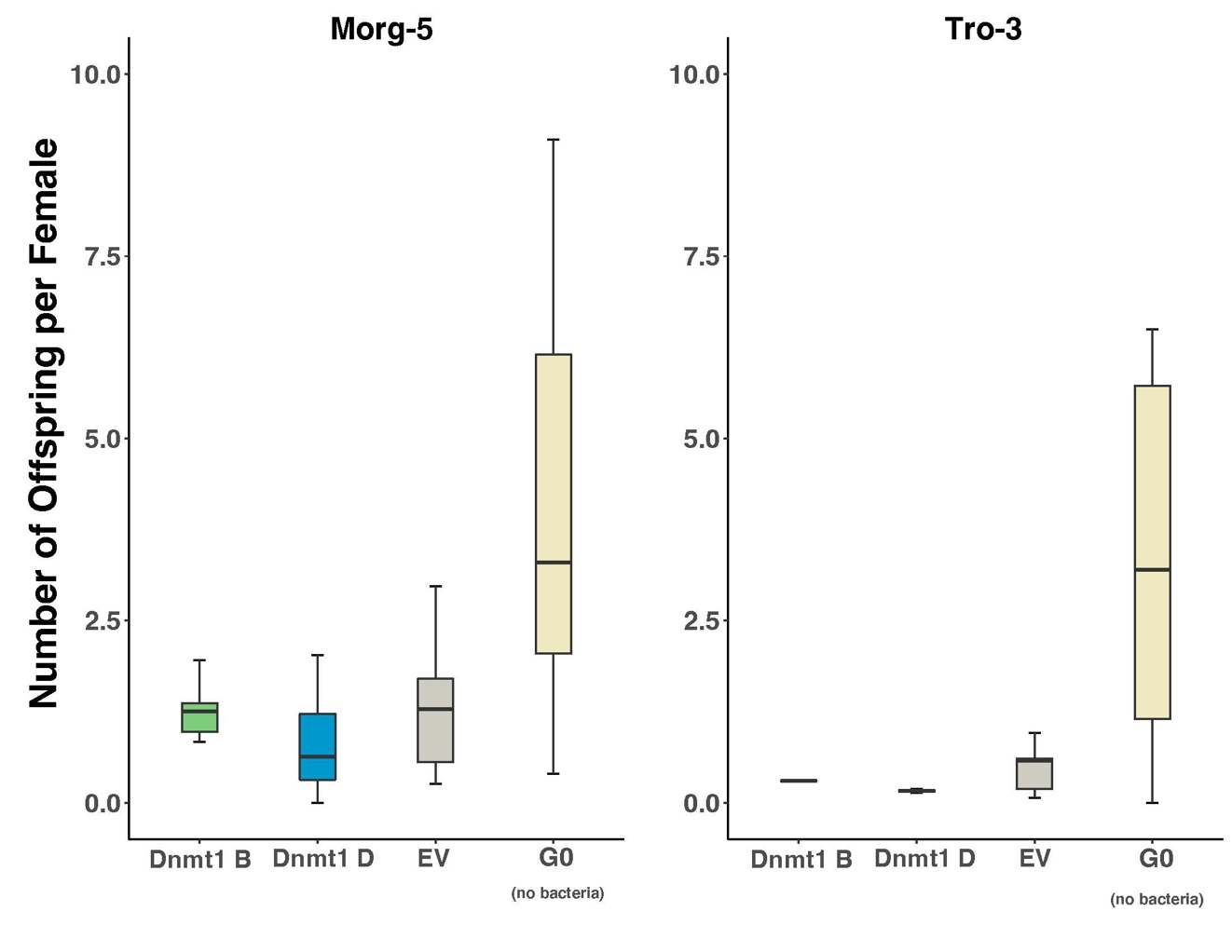


**Supplemental Figure 4.** Standardized boxplots of the number of neonates produced each day, total number of neonates for each treatment divided by the number of surviving females. Animals treated with the DNMT1 B vector are shown in green, DNMT1 D vector in blue, and EV in gray. No significant difference was detected between vector treatments. When compared to the maternal generation, G_0_, all three vector treatments in both clones produced significantly fewer offspring over a similar timeframe.


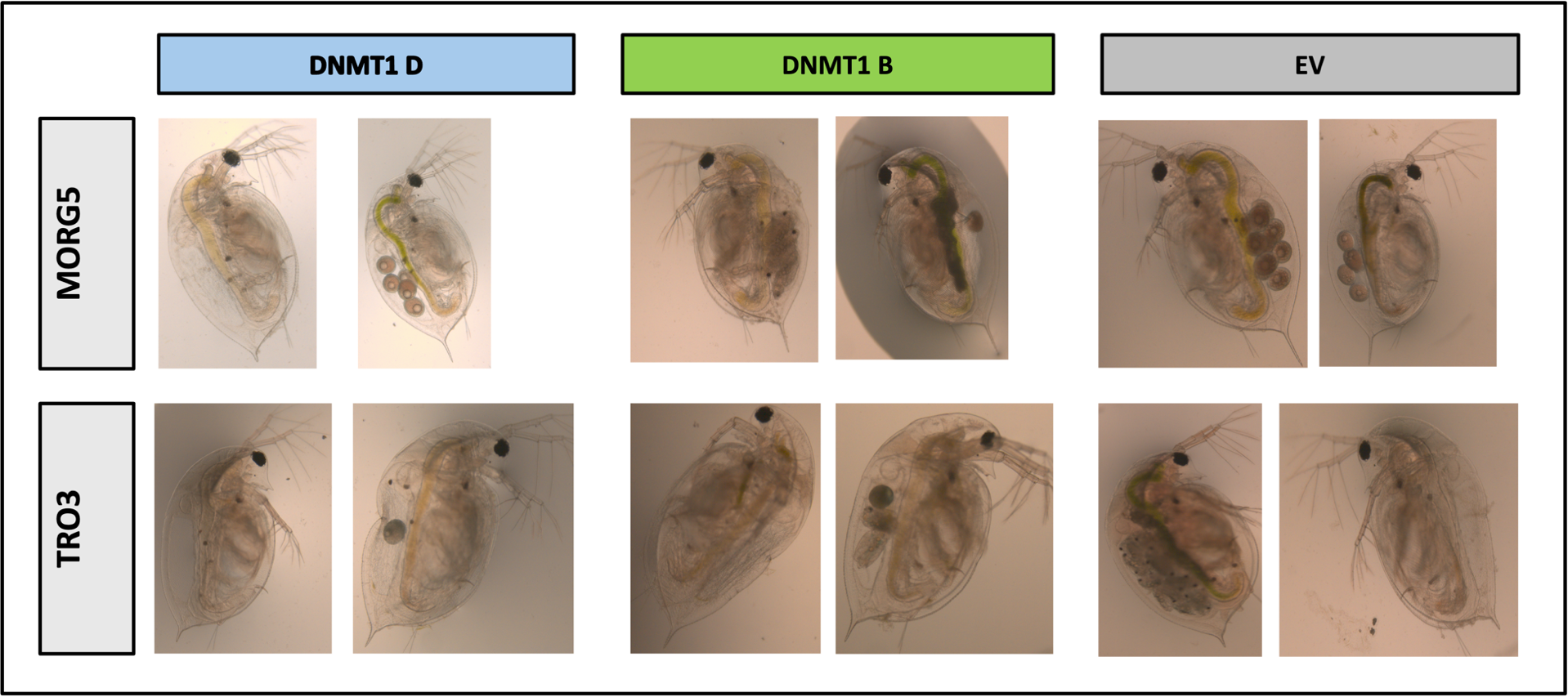


**Supplemental** **Figure 5.** Photographs taken on day 10 of the RNAi bacterial feeding regime. TRO3 animals show more obvious signs of stress: loss of pigmentation in the carapace, dark yellow to brown coloring in the gut, decreased egg production (black arrows), and a deformity in the carapace (blue arrows). MORG5 animals do not show the same signs of distress in any treatment. Vitellogenesis (red arrow) and egg production continued unimpaired (orange arrows), gut coloring indicated constant filter feeding (green arrows), and no physical deformities were observed.


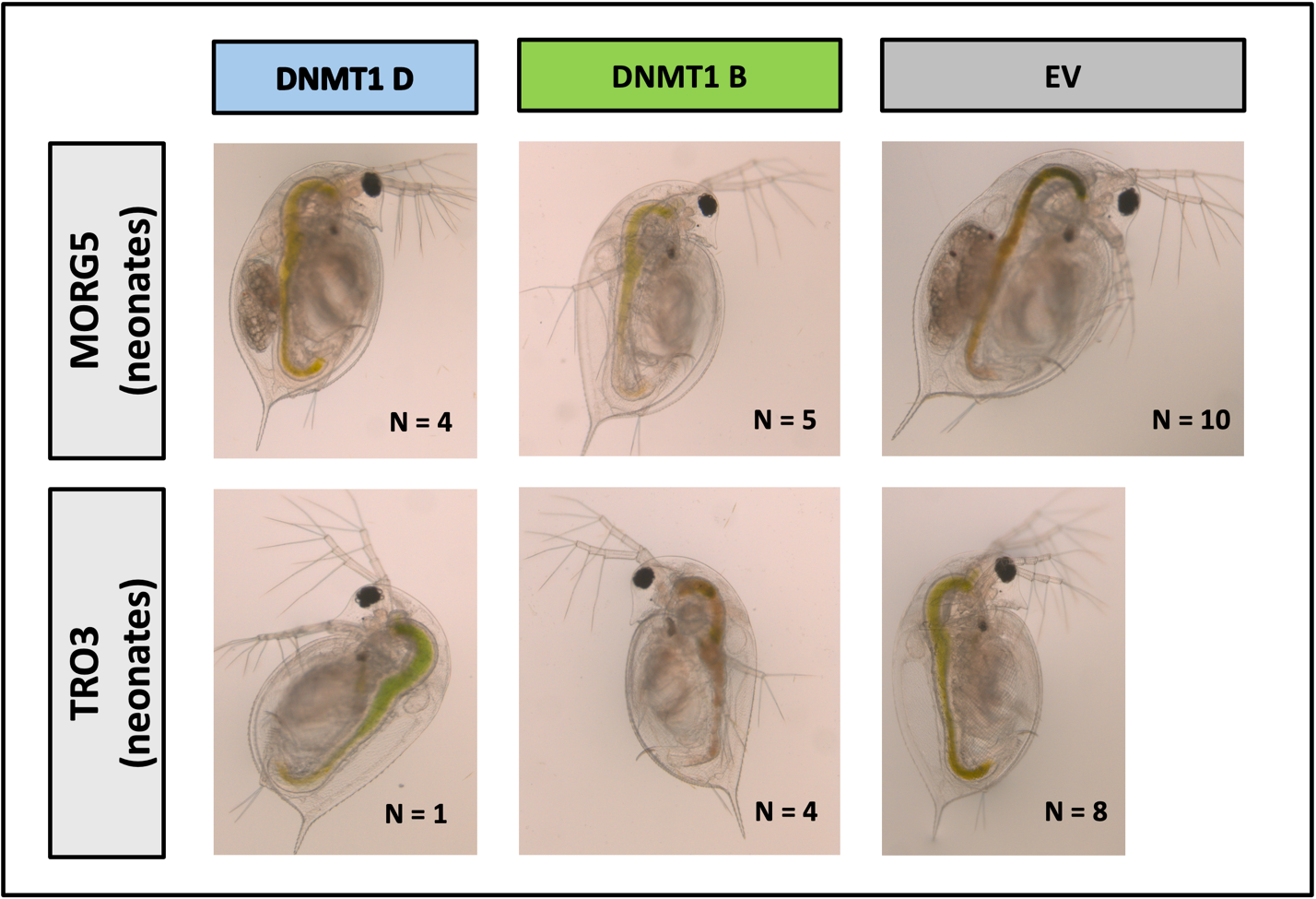


**Supplemental** **Figure 6.** Neonates born into during the bacterial feeding regime were collected (n = 10 per treatment per clone) and treated with the same vector as their mother. The N represents how many of the original cohort survived by day 12 when the photographs were taken. Developmentally, all surviving offspring appear to have defined brood chambers but only MORG5, DNMT1 D, and EV produced eggs. Maturation was delayed in both treatments by approximately 2.5 days.


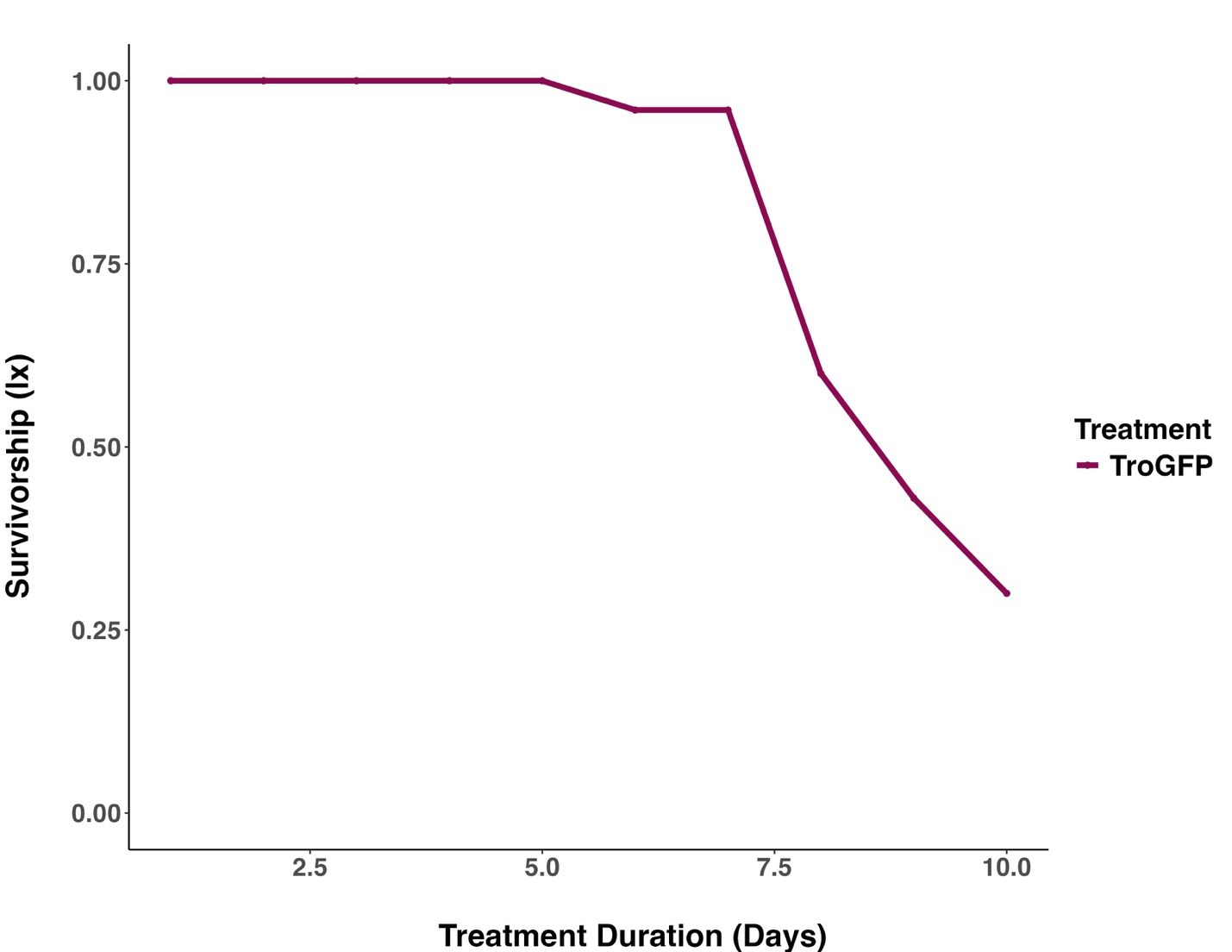


**Supplemental Figure 7.** Plot showing the effects of the GFP treatment on survivorship (lx) of TRO3 animals. TRO3 GFP survivorship was significantly reduced ($\chi$^2^ = 14.046, p < 0.029, *df* = 6) when compared with animals from Experiment 2.


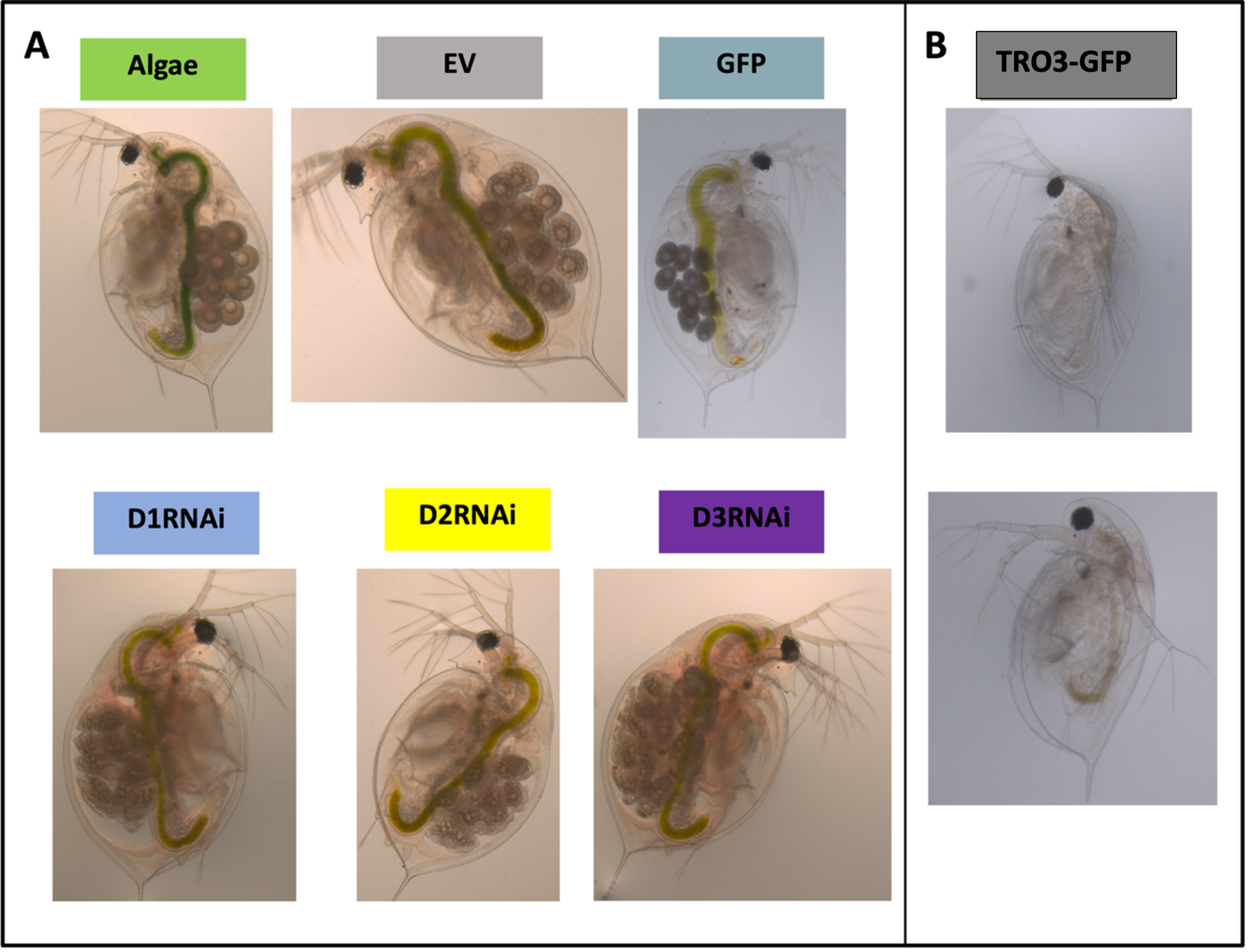


**Supplemental Figure 8.** Photographs taken on day 10 of the RNAi bacterial feeding regime. **A)** Photographs of the MORG5 clone from each treatment Algae (green), Empty Vector (grey), L4440 with GFP (turquoise), and each DNA methyltransferase RNAi treatment 1 (blue), 2 (yellow), and 3 (purple). On days 2 and 3, animals from all three DNMT RNAi treatments experienced two ecdysis events, both premature and without the release of neonates. Gut coloring for all six treatments is light to bright green, indicating constant filter feeding of fresh algae. Vitellogenesis and egg production continued unimpaired with normal egg development. All three DNMT RNAi treatments resulted in animals producing high levels of hemoglobin, giving them a pink coloring. **B)** Photographs of surviving TRO3 animals from the GFP plasmid vector treatment. Animals appear translucent; gut coloring is either dark yellow-brown or clear, indicating inconsistent, minimal filtering or a complete halt to filter feeding. Despite reaching maturation at the start of the experiment, no offspring were produced, and vitellogenesis was not observed during the 10-day feeding regime.


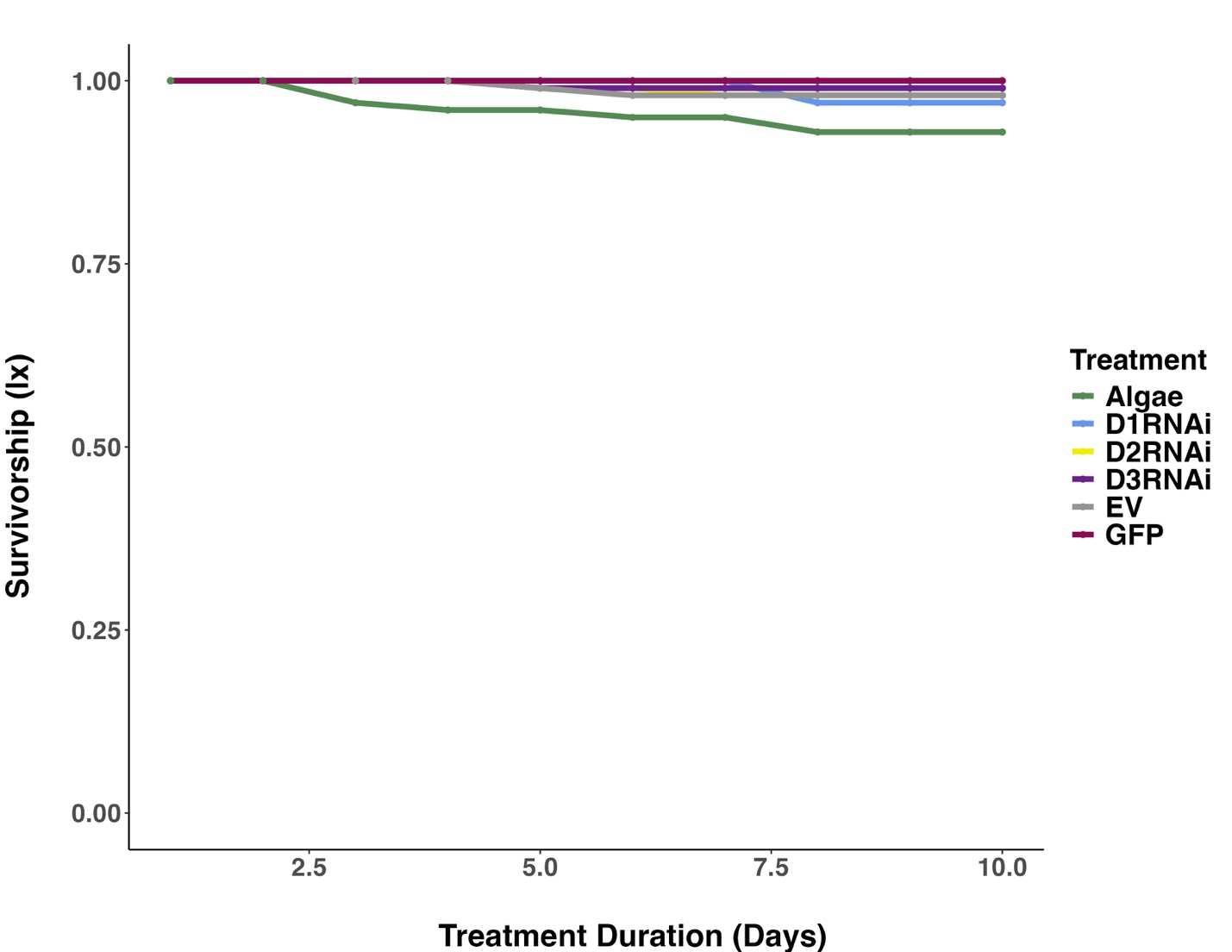


**Supplemental Figure 9.** Plot showing the effects of each treatment on survivorship (*lx*) for the MORG5 clone. Treatment identification is as follows: Algae (green), Empty Vector (grey), L4440 with GFP (pink), and each DNA methyltransferase RNAi treatment 1 (blue), 2 (yellow), and 3 (purple). The was no significant effect of treatment on survivorship observed for the MORG5 clone ($\chi$^2^ = 0.366, p = 0.996, *df* = 5).

**Supplemental Figure 10.** Magnitude of on- and off-target expression reduction indicated by line thickness. Curved arrows indicate that DNMT RNAi vector treatment on the DNMT of interest. Straight arrows indicate the direction of the off-target effect of each DNMT RNAi vector treatment on other DNMTs.

| **Supplemental Table 1: DNMT1 Dunn’s Test Multiple Comparison**  Kruskal-Wallis: $\chi$^2^ = 28.99, *df* = 5, p < 0.0001 | | | | | |
| --- | --- | --- | --- | --- | --- |
|  | Algae | D1RNAi | D2RNAi | D3RNAi | EV |
| D1RNAi | -2.334  **0.042** |  |  |  |  |
| D2RNAi | -2.453  **0.053** | -0.119  0.905 |  |  |  |
| D3RNAi | -3.089  **0.01** | -0.745  0.563 | -0.636  0.562 |  |  |
| EV | -0.634  0.606 | 1.698  0.134 | 1.817  0.115 | 2.453  **0.043** |  |
| GFP | -4.717  **0.000** | -2.383  **0.043** | -2.264  **0.044** | -1.628  0.141 | -4.081  **0.000** |

Dunn’s pairwise *z* statistic and p-value, significant p-values are in bold

Alpha = 0.05

Method = Benjamini – Hochberg

| **Supplemental Table 2: DNMT2 Dunn’s Test Multiple Comparison**  Kruskal-Wallis: $\chi$^2^ = 23.27, *df* = 5, p < 0.0002 | | | | | |
| --- | --- | --- | --- | --- | --- |
|  | Algae | D1RNAi | D2RNAi | D3RNAi | EV |
| D1RNAi | -4.004  **0.000** |  |  |  |  |
| D2RNAi | -2.516  **0.035** | 1.489  0.256 |  |  |  |
| D3RNAi | -3.012  **0.013** | 0.992  0.438 | -0.496  0.775 |  |  |
| EV | -2.600  **0.035** | 1.405  0.267 | -0.084  0.933 | 0.412  0.785 |  |
| GFP | -4.283  **0.000** | -0.280  0.836 | -1.768  0.193 | -1.272  0.305 | -1.684  0.197 |

Dunn’s pairwise *z* statistic and p-value, significant p-values are in bold

Alpha = 0.05

Method = Benjamini – Hochberg

| **Supplemental Table 3: DNMT3 Dunn’s Test Multiple Comparison**  Kruskal-Wallis: $\chi$^2^ = 34.77, *df* = 5, p < 0.0001 | | | | | |
| --- | --- | --- | --- | --- | --- |
|  | Algae | D1RNAi | D2RNAi | D3RNAi | EV |
| D1RNAi | -2.921  **0.011** |  |  |  |  |
| D2RNAi | -2.348  **0.035** | 0.573  0.708 |  |  |  |
| D3RNAi | -2.719  **0.014** | 0.203  0.839 | -0.370  0.821 |  |  |
| EV | -2.055  **0.067** | 0.867  0.579 | 0.294  0.824 | 0.664  0.691 |  |
| GFP | -5.787  **0.000** | -2.865  **0.010** | -3.439  **0.003** | -3.068  **0.008** | -3.732  **0.001** |

Dunn’s pairwise *z* statistic and p-value, significant p-values are in bold

Alpha = 0.05

Method = Benjamini – Hochberg
